# Prognostic Implications of Metabolism Related Gene Signature in Cutaneous Melanoma

**DOI:** 10.3389/fonc.2020.01710

**Published:** 2020-09-09

**Authors:** Furong Zeng, Juan Su, Cong Peng, Mengting Liao, Shuang Zhao, Ying Guo, Xiang Chen, Guangtong Deng

**Affiliations:** ^1^Hunan Key Laboratory of Skin Cancer and Psoriasis, Department of Dermatology, Hunan Engineering Research Center of Skin Health and Disease, Xiangya Hospital, Central South University, Changsha, China; ^2^National Clinical Research Center for Geriatric Disorders, Xiangya Hospital, Central South University, Changsha, China

**Keywords:** melanoma, metabolism related genes, overall survival, prognosis, nomogram

## Abstract

Metabolic reprogramming is closely related to melanoma. However, the prognostic role of metabolism-related genes (MRGs) remains to be elucidated. We aimed to establish a nomogram by combining MRGs signature and clinicopathological factors to predict melanoma prognosis. Eighteen prognostic MRGs between melanoma and normal samples were identified using The Cancer Genome Atlas (TCGA) and GSE15605. *WARS* (HR = 0.881, 95% CI = 0.788–0.984, *P* = 0.025) and *MGST1* (HR = 1.124, 95% CI = 1.007–1.255, *P* = 0.037) were ultimately identified as independent prognostic MRGs with LASSO regression and multivariate Cox regression. The MRGs signature was established according to these two genes and externally validated in the Gene Expression Omnibus (GEO) dataset. Kaplan-Meier survival analysis indicated that patients in the high-risk group had significantly poorer overall survival (OS) than those in the low-risk group. Furthermore, the MRGs signature was identified as an independent prognostic factor for melanoma survival. An MRGs nomogram based on the MRGs signature and clinicopathological factors was developed in TCGA cohort and validated in the GEO dataset. Calibration plots showed good consistency between the prediction of nomogram and actual observation. The receiver operating characteristic curve and decision curve analysis indicated that MRGs nomogram had better OS prediction and clinical net benefit than the stage system. To our knowledge, we are the first to develop a prognostic nomogram based on MRGs signature with better predictive power than the current staging system, which could assist individualized prognosis prediction and improve treatment.

## Introduction

Cutaneous melanoma (hereafter “melanoma”), a tumor most commonly observed in fair-skin populations, is the most lethal form of skin malignancy with great heterogeneity. Its incidence has been increasing worldwide over recent decades (1), and the prognosis of melanoma patients is poor due to its invasiveness and metastasis (2). Numerous efforts have been made to develop useful tools for melanoma prognosis predictions. The most frequently used tool is the American Joint Committee on Cancer's staging system for tumor-node-metastasis, but it is not satisfactory in current clinical practice. Increasing studies show that patients differed considerably in prognosis even at the same tumor-node-metastasis stage due to the discrepant genetic backgrounds (3). Therefore, it is still necessary to explore novel melanoma prognostic biomarkers for optimal therapeutic strategies.

Metabolic reprogramming, an emerging hallmark of cancer, allows cancer cells to survive, proliferate, and disseminate (4, 5). In the 1920s, Otto Warburg observed that proliferating ascites tumor cells preferentially performed glycolysis, even in oxygen-rich circumstances (6). This seminal finding has been observed in a wide variety of cancers and currently has been exploited clinically using 18F-deoxyglucose positron emission tomography (7). Mechanically, in proliferating tumor cells, glycolysis, instead of pure mitochondrial metabolism, could provide essential intermediates for biosynthetic pathways, such as lipid or nucleotide synthesis (8). Emerging studies highlight the close association between melanoma and metabolic reprogramming. For example, 18F-deoxyglucose positron emission tomography was applied for the detection of the early response to the B-Raf proto-oncogene, serine/threonine kinase (*BRAF*) inhibitor, vemurafenib, in *BRAF*-mutant melanoma patients (9). Also, some potential drugs navigating metabolic pathways have been exploited for melanoma in preclinical or clinical scenarios (10). Therefore, metabolism-related genes (MRGs) are promising therapeutic targets and prognostic predictors in melanoma.

Nomogram has become a reliable and convenient tool in cancer prognosis predictions (11, 12). Several prognostic nomograms have been established for predicting the prognosis of melanoma in recent years (13–15), while global expression pattern based on MRGs has not previously been recognized in melanoma. In this study, we aimed to develop and validate a novel prognostic nomogram based on MRGs signature and clinicopathological factors for ideally predicting the prognosis of melanoma patients.

## Materials and Methods

### Acquisition of MRGs

MRGs were extracted from all 41 metabolic pathways in the Kyoto Encyclopedia of Gene and Genomes (KEGG) pathway (c2.cp.kegg.v7.0.symbols.gmt) from the Gene Set Enrichment Analysis (GSEA) website (https://www.gsea-msigdb.org/gsea/downloads.jsp#msigdb). Finally, a total of 948 MRGs were identified for our study.

### Data Retrieval and Processing

The training cohort dataset with 460 melanoma RNA-sequencing data and clinical information was obtained from The Cancer Genome Atlas (TCGA) (https://portal.gdc.cancer.gov/). GSE15605 and GSE54467 were derived from the Gene Expression Omnibus (GEO) database (https://www.ncbi.nlm.nih.gov/geo/). GSE15605, which included 46 primary melanoma samples and 16 normal skin samples, was used to identify differentially expressed genes (DEGs) using GEO2R. An adjusted *P* < 0.01 and a |log_2_ (FC) |> 2 were considered the cutoffs for identifying DEGs. GSE54467, which included 79 melanoma patients, was used as the GEO validation dataset. The intersected genes in TCGA cohort and GSE54467 dataset were extracted, and their expressions were normalized using the “limma” and “sva” packages using R software version 3.6.0. MRGs in these intersected genes were used for the following univariate Cox regression analysis. Patient clinical and pathological characteristics in TCGA and GEO cohorts are summarized in Supplementary Table 1.

### Construction and Validation of the Prognostic MRGs Signature

A univariate Cox regression analysis was performed to screen out the prognosis related MRGs. Then the prognosis related MRGs were overlapped with the DEGs to obtain the prognostic MRGs. The least absolute shrinkage and selection operator (LASSO) regression analysis with tenfold cross-validation was subsequently applied by using “glmnet” and “survival” packages (16). The independent prognostic MRGs were generated through a multivariate Cox regression analysis and used to construct the prognostic MRGs signature with the following formula: *Risk score* = (β1 × *expression of MRG*1)+ (β2 × *expression of MRG*2)+ ⋯ + (β*n* × *expression MRGn*). Patients were divided into high-risk and low-risk groups according to the median risk score. Kaplan-Meier survival analysis was performed to evaluate the association between the prognostic MRGs signature and overall survival (OS) in melanoma patients. Moreover, univariate and multivariate Cox regression was used to identify the independent prognostic factors, including age, stage, and MRGs signature. The prognostic MRGs signature was externally validated in the GEO dataset and calculated with the same formula and cutoff. *P* < 0.05 was regarded as statistically significant.

### Functional Enrichment Analysis

Gene ontology and KEGG pathway analyses were performed for the differentially expressed MRGs using “org.Hs.eg.db,” “clusterProfiler,” “enrichplot,” “ggplot2,” and “GOplot” packages in R. The adjusted *P* < 0.05 was considered statistically significant.

### Validation of the Independent Prognostic MRGs

Gene Expression Profiling Interactive Analysis (GEPIA) is a web-based tool to analyze gene expression and function based on the RNA-seq data from TCGA (one normal sample and 460 melanoma samples) and Genotype-Tissue Expression (GETx) (557 normal samples). The differential expression of these independent prognostic MRGs was verified using GEPIA. Their expression of *WARS* and *MGST1* were validated using clinical specimens from the Human Protein Atlas (HPA) database (http://www.proteinatlas.org). A Kaplan-Meier survival analysis was conducted to validate the prognostic value of the independent prognostic MRGs using GEPIA.

### GSEA

GSEA was performed in java GSEA (version 4.0.3) based on the Molecular Signatures Database version 6.2. Through comparing the high- and low- risk groups in 460 melanoma patients from TCGA dataset. C2 (curated gene sets), C5 (GO gene sets), and C6 (Oncogenic signature) were searched to identify enriched KEGG pathways, biological processes, cellular components, molecular functions, and oncogenic signatures. FDR q < 0.05, |NES| > 1 were considered statistically significant.

### Construction and Validation of the Nomogram

All the independent prognostic factors were enrolled to establish a nomogram in TCGA training cohort. A calibration curve was plotted to evaluate the consistency between the nomogram and actual observation. The concordance index (C index) and the area under the curve (AUC) in receiver operating characteristic (ROC) curves were applied to assess the predictive accuracy. Decision curve analysis, an approach to assess the clinical value of models by integrating the preferences of the patients into the analysis, was used to evaluate the clinical benefits of stages and our nomogram to facilitate decisions about test selection and use (17).

## Results

### *WARS* and *MGST1* Were the Independent Prognostic MRGs

**Figure 1:**
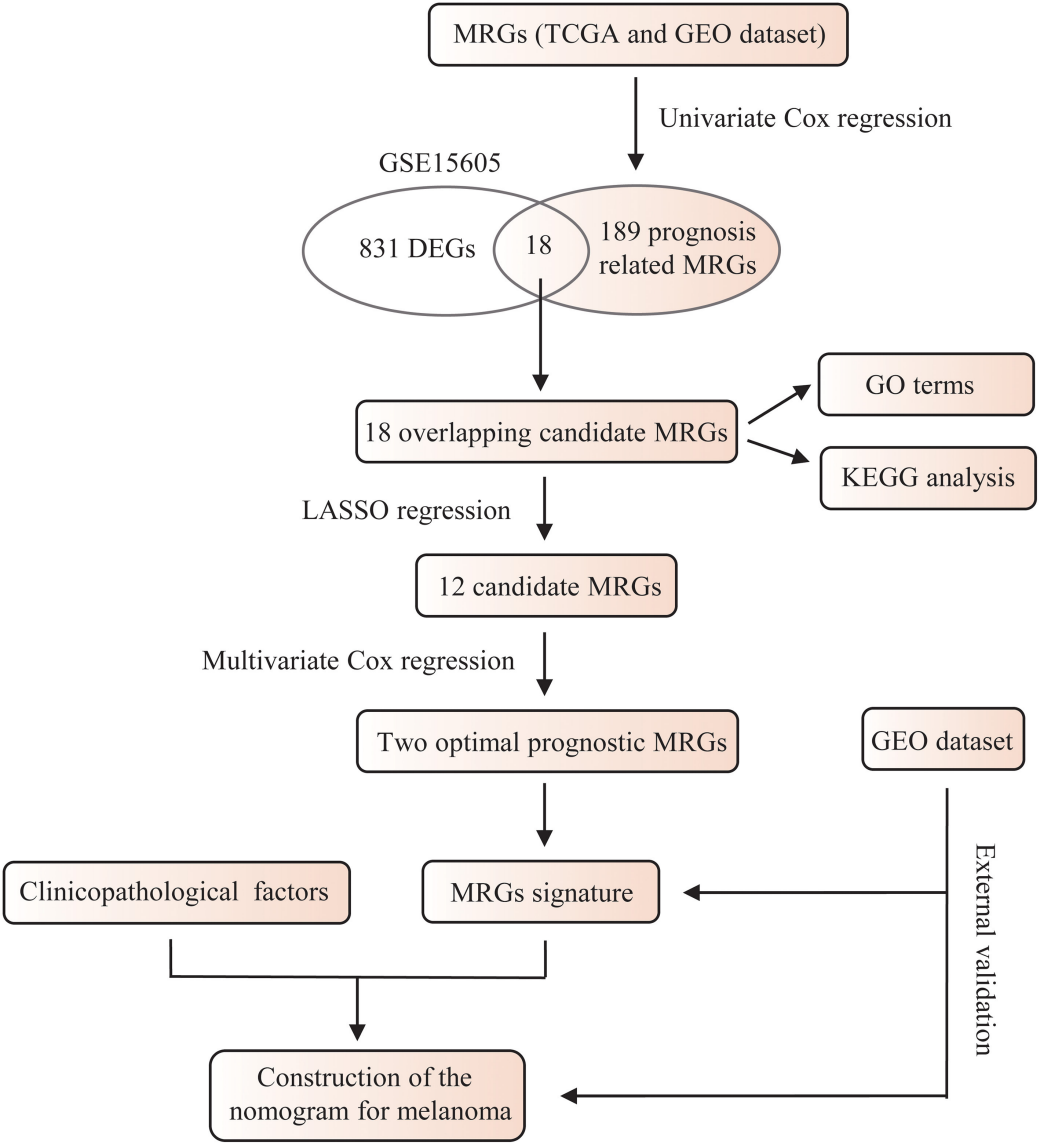
A flowchart of the study procedure.

**Figure 2:**
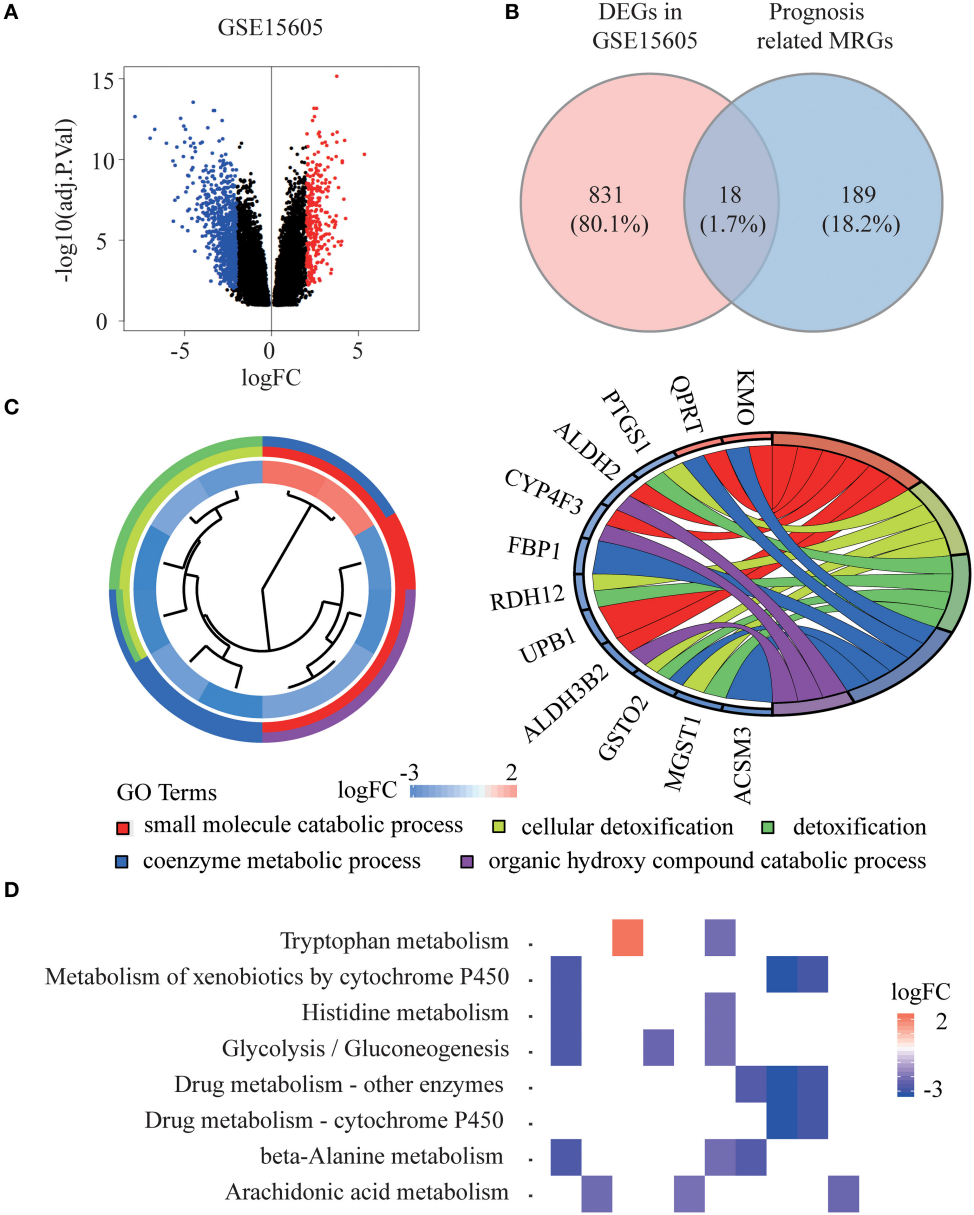
Identification of prognostic metabolism related genes (MRGs) and functional enrichment analysis. **(A)** Volcano plot of differentially expressed genes (DEGs) between melanoma and normal samples of GSE15605 dataset. The red dots represent up-regulated genes, and the green dots represent down-regulated genes (adj. *P* < 0.01 and |log_2_ (FC) | > 2). **(B)** Venn diagram showing the intersection of the DEGs in GSE15605 and prognosis related MRGs. **(C,D)** Gene ontology (GO) terms **(C)** and Kyoto Encyclopedia of Gene and Genomes (KEGG) pathways **(D)** of 18 prognostic MRGs.

**Figure 3:**
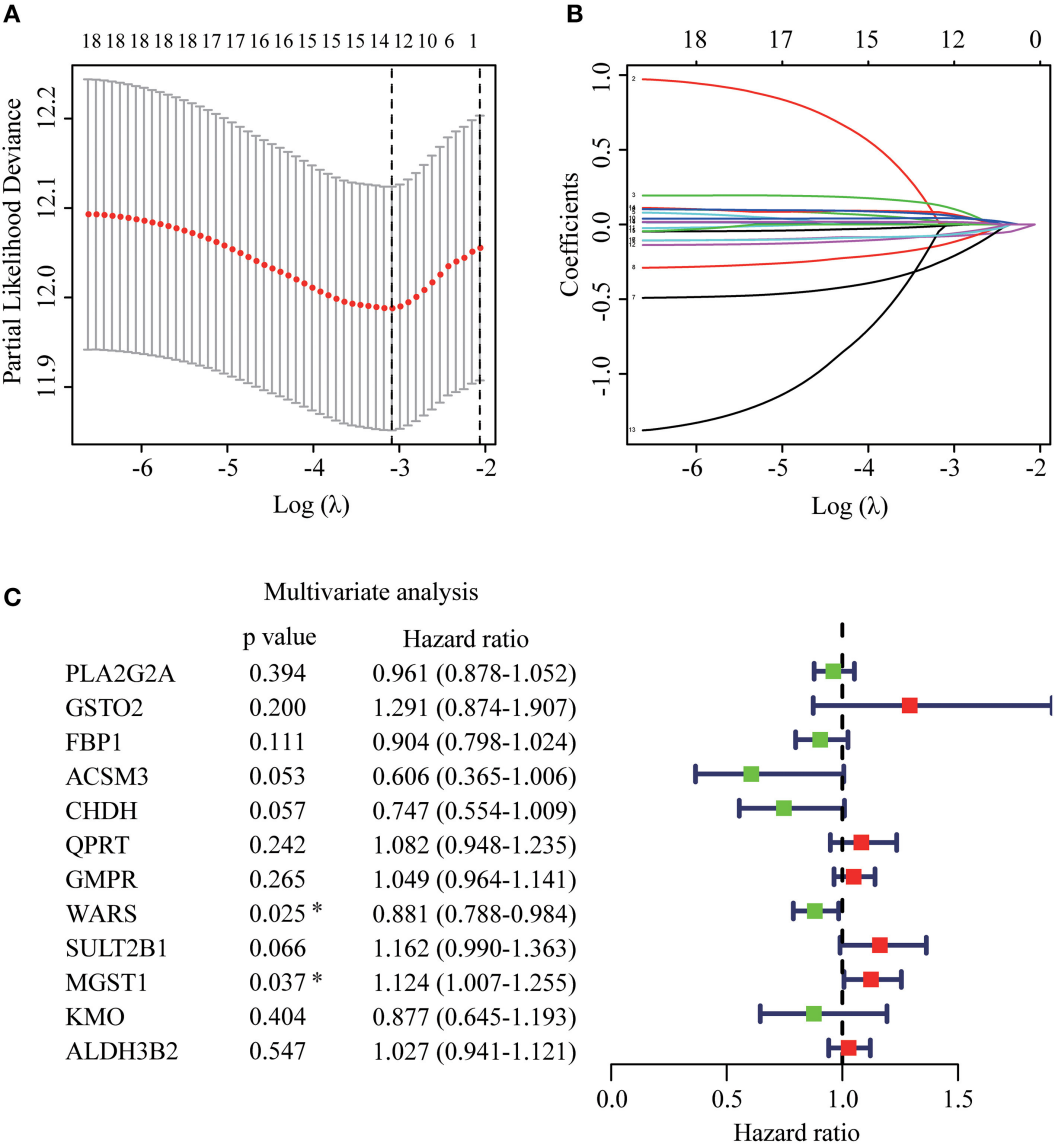
Establishment of prognostic MRGs signature. **(A)** Selection of the optimal parameter in the least absolute shrinkage and selection operator (LASSO) regression with tenfold cross-validation. **(B)** LASSO coefficient profiles of the candidate prognosis related MRGs. **(C)** Multivariate Cox regression analysis of 12 candidate prognosis related MRGs.

The whole flowchart for the study procedure is presented in Figure 1. A total of 849 DEGs were found in GSE15605 by volcano plot (*P* ≤ 0.01, |log_2_FC| ≥ 2; Figure 2A). Using univariate Cox regression, 207 MRGs associated with OS were identified in TCGA training cohort (Supplementary Table 2). Differentially expressed MRGs were the intersection of the above two gene sets, and finally, 18 overlapping prognostic MRGs were obtained (Figure 2B). Gene ontology functional enrichment and KEGG analyses were performed on the prognostic MRGs (Figures 2C,D). Gene ontology enrichment analysis showed that these DEGs were mainly enriched in small molecule catabolic processes, cellular detoxification, and detoxification. KEGG analysis revealed that the DEGs were mainly enriched in tryptophan metabolism, metabolism of xenobiotics by cytochrome P450, and histidine metabolism. To avoid collinearity, the differentially expressed MRGs were entered into a LASSO regression with ten-fold cross-validation, and 12 candidate MRGs were ultimately selected (Figures 3A,B). Then, multivariate Cox regression was applied and results showed that tryptophanyl-tRNA synthetase 1 (*WARS*) (HR = 0.881, 95% CI = 0.788–0.984, *P* = 0.025) and microsomal glutathione S-transferase 1 (*MGST1*) (HR = 1.124, 95% CI = 1.007–1.255, *P* = 0.037) were the independent prognostic MRGs (Figure 3C).

### Verification of *WARS* and *MGST1* Expression and Prognosis

**Figure 4:**
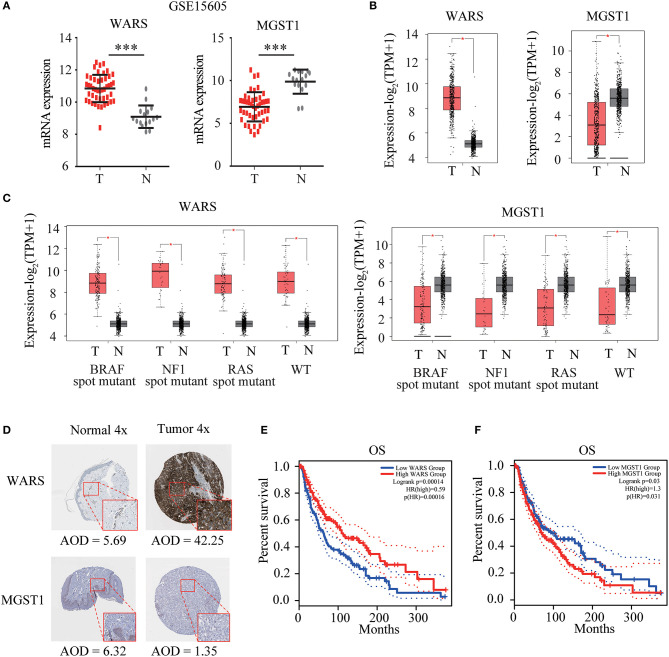
Verification of *WARS* and *MGST1*. **(A)** The expression of *WARS* and *MGST1* between melanoma and normal skin in GSE15605. *N* (T) = 46 and *N* (N) = 16. ***P < 0.001. **(B)** The expression of *WARS* and *MGST1* between melanoma and normal skin using Gene Expression Profiling Interactive Analysis (GEPIA) database. The number of sorts: *N* (T) = 460 and *N* (N) = 558. **(C)** The expression of *WARS* and *MGST1* in three mutational signatures (BRAF, NF1 and RAS) and wild types (WT) of melanoma. The number of sorts: N (T) = 147 and N (N) = 558 in BRAF mutation; N (T) = 27 and N (N) = 558 in NF1 mutation; N (T) = 91 and N (N) = 558 in RAS mutation; N (T) = 47 and N (N) = 558 in WT. **(D)** The representative protein expressions of *WARS* and *MGST1* between normal and melanoma tissues in the Human Protein Atlas database (http://www.proteinatlas.org). AOD, average optical density, calculated by integrated optical density/area. The expressions were quantified by Image J (version 1.52a). **(E,F)** Kaplan-Meier curves for overall survival (OS) of *WARS* **(E)** and *MGST1* **(F)** in melanoma patients using GEPIA. N (high) = 229, N (low) = 229. T, tumor; N, normal skin. *P < 0.05.

*WARS* and *MGST1* were highly expressed and downregulated in GSE15605 melanoma datasets, separately (Figure 4A). The differential expression of these two genes was further validated in the GEPIA database (Figure 4B). Interestingly, their expressions were independent of the status of key melanoma mutations, including *BRAF*, neurofibromin 1 (*NF1*), and *RAS* mutations and triple wild type in melanoma (Figure 4C). Moreover, the protein level encoded by these two genes was consistent with their gene expression using the HPA website. *WARS* was strongly positive in melanoma tissue, while *MGST1* was weakly positive in normal skin tissue (Figure 4D). Kaplan-Meier survival curves were further conducted to evaluate the prognostic value of each gene. Though *WARS* and *MGST1* were not associated with disease free survival (Figure S1), we arrived at the same conclusion that *WARS* was a protective gene (HR = 0.59, *P* < 0.001), while *MGST1* was a risk gene (HR = 1.3, *P* = 0.031) for OS in melanoma (Figures 4E,F).

### MRGs Signature Acts as an Independent Prognostic Predictor

**Figure 5:**
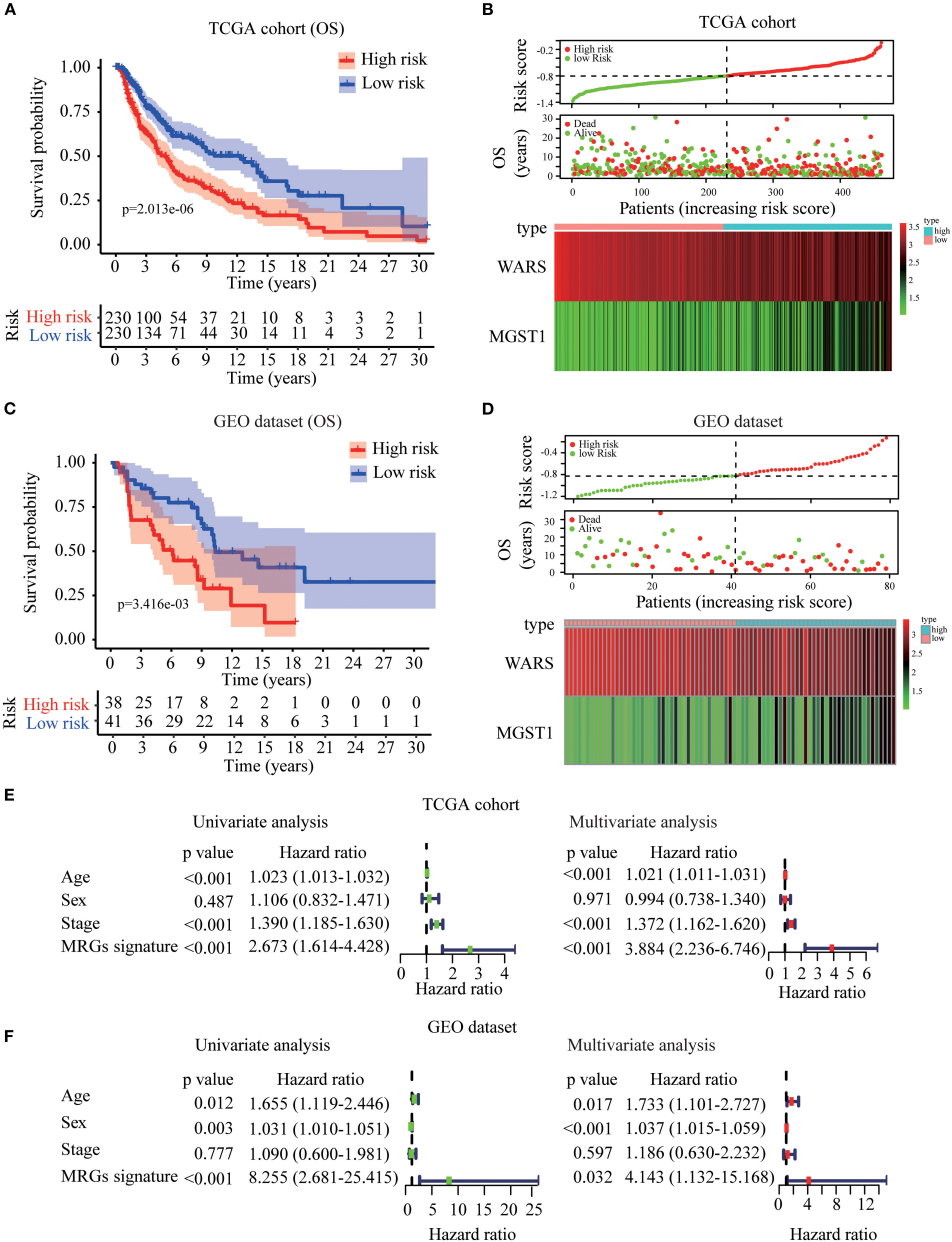
Construction and validation of MRGs signature in melanoma. **(A–D)** Kaplan-Meier analysis for OS based on the MRGs signature of melanoma patients in TCGA training cohort **(A)** and GEO validation dataset **(C)**. The distribution of risk score, survival status and expression heatmap of the two MRGs in TCGA training cohort **(B)** and GEO validation dataset **(D)**. **(E,F)** Cox regression analysis of MRGs signature and clinicopathological risk factors in TCGA training cohort **(E)** and GEO validation dataset **(F)**.

Based on *WARS* and *MGST1*, MRGs signature was established to predict melanoma prognosis according to the formula: *MRGs signature* = (−0.139 × *expression of WARS*) + (0.122 × *expression of MGST*1). The prognostic signature for each patient in TCGA training cohort was calculated. All patients were divided into high-risk or the low-risk groups using the median signature as the cutoff (−0.804). The result demonstrated that patients with higher risk scores had worse OS than those in the low-risk group (Figure 5A). The distributions of risk score, survival status, and a heatmap of the gene expression profile are presented in Figure 5B. Interestingly, in our MRGs signature, disease free survival was also much shorter in the high-risk group (Figure S2), suggesting that the MRGs signature is a better predictor than individual gene. To examine the robustness of the MRGs signature, we used a GEO dataset to externally validate the prognostic value of the model. The same signature formula and the cutoff were applied to classify the melanoma patients into the high-risk group (*n* = 38) and low-risk group (*n* = 41) in the GEO validation dataset. Consistently, the results showed that patients in the high-risk group generally had increased *MGST1*, decreased *WARS*, and worse overall survival than those in the low-risk group (Figures 5C,D). To determine whether MRGs signature could act as an independent prognostic factor, MRGs signature and clinicopathological factors including age, sex, and stage were entered into a univariate Cox regression analysis, indicating that the MRGs signature was significantly associated with OS (HR = 2.673, 95% CI = 1.614–4.428, *P* < 0.001; Figure 5E). A multivariate Cox regression analysis revealed that the MRGs signature was an independent prognostic factor (HR = 3.884, 95% CI = 2.236–6.746, *P* < 0.001; Figure 5E). These results were consistent in the GEO dataset (univariate Cox regression analysis: HR = 8.255, 95% CI = 2.681–25.415, *P* < 0.001; multivariate Cox regression analysis: HR = 4.143, 95% CI = 1.132–15.168, *P* < 0.001; Figure 5F).

### GSEA for MRGs Signature

To identify the underlying molecular mechanism of the MRGs signature, we conducted GSEA to compare the high- and low- risk groups in 460 melanoma patients from TCGA dataset. In the high-risk group, no GO terms, KEGG pathways or oncologic signatures were significantly enriched. However, in the low-risk group, 574 GO terms were significantly enriched especially in regulation of type I interferon production, NF-κB pathway and regulation of autophagy Figure S3A. 20 KEGG pathways highlighted that antigen processing and presentation, apoptosis, and JAK/STAT signaling pathways were enriched in the low-risk group Figure S3B. Moreover, 14 oncogenic signatures were significantly enriched in low-risk group including the CAMP, MEK, P53, and other pathways Figure S3C. These significant terms in each module were summarized in Table S3.

### Construction and Validation of MRGs Nomogram

**Figure 6:**
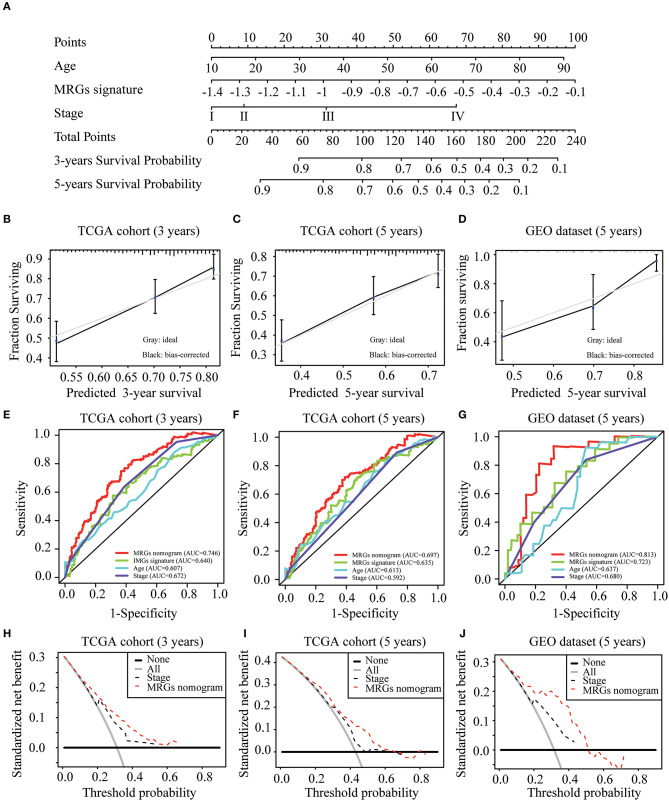
Development and validation of MRGs nomogram. **(A)** Development of MRGs nomogram. **(B–D)** Calibration plots for predicting 3-years **(B)**, 5-years **(C)** OS in the TCGA training cohort and 5-years OS in the GEO validation dataset **(D)**. **(E–G)** Receiver operating characteristic (ROC) curves of the MRGs nomogram, MRGs signature, age and stage at 3-years **(E)** and 5-years **(F)** OS in the TCGA training cohort and 5-years OS in the GEO validation dataset **(G)**. **(H–J)** Decision curve analysis of the MRGs nomogram and stage at 3-years **(H)** and 5-years **(I)** prediction in the TCGA training cohort and 5-years prediction in the GEO validation dataset **(J)**.

To construct a clinically applicable method for predicting the prognosis of melanoma patients, independent prognostic predictors including age, stage, and MRGs signature were enrolled to establish a nomogram to predict the survival probability at 3 and 5 years based on TCGA training cohort (Figure 6A). The calibration plots (Figures 6B,C) showed an excellent match with the ideal curve at 3- and 5-years survival rates in TCGA training cohort. In the validation dataset, the calibration plots also showed good agreement between the predicted and actual outcome of 5-years OS rates (Figure 6D). The C index of the nomogram was 0.707 in TCGA training cohort. Moreover, the ROC curve showed a more favorable predictive ability for the 3-years OS rates (AUC = 0.746) as compared to MRGs signature (AUC = 0.640), age (AUC = 0.607), and stage (AUC = 0.672; Figure 6E), as well as for the 5-years OS rates (AUC = 0.697) as compared to MRGs signature (AUC = 0.635), age (AUC = 0.613), and stage (AUC = 0.592; Figure 6F). In the validation dataset, the C index of the nomogram for predicting OS was 0.730. The nomogram also has the largest discrimination ability (AUC = 0.813) as compared to MRGs signature (AUC = 0.723), age (AUC = 0.637), and stage (AUC = 0.680) for 5-years OS rates (Figure 6G). Decision curve analysis results in both TCGA training cohort and the GEO validation dataset suggested that our nomogram could be more beneficial than traditional stages in predicting the survival for melanoma patients (Figures 6H–J).

## Discussion

Altered metabolism is considered to be related to cancer cell survival and growth (4, 18). Various metabolisms, such as the glucose and glutamine metabolism of cancer cells, can be significantly changed by tumor microenvironment across an individual tumor (19, 20). However, the tumor can also acclimatize itself to metabolic reprogramming, suggesting the specificity of metabolic targets to each cancer (20). Metabolic gene signatures have been shown to have a prognostic role in cancers (21, 22). Melanoma is a type of tumor highly related to metabolic reprogramming, including glycolysis, protein/amino acid metabolism, and lipid metabolism (23). The melanoma cells need to increase oxidative stress and undergo metabolic changes during metastasis (24). A recent study showed that metabolic differences among melanoma cells conferred differences in metastatic potential, which was due to the differences in the function of the *MCT1* transporter (25). All these studies highlight the potential value of generating a metabolism-related model for the prognosis prediction of melanoma.

In the present study, we first identified 207 metabolism-related genes, based on TCGA, significantly correlated with prognosis in the univariate Cox regression analysis. In GSE15605, which contains the largest normal samples in the GEO database, 849 DEGs were identified by a volcano plot. Then the intersected genes between DEGs and prognostic MRGs were entered into a LASSO regression and multivariate Cox regression. Ultimately, MRGs signature, including *WARS* and *MGST1*, were obtained. According to the median risk score of MRGs signature, 460 melanoma patients in TCGA were divided into the high- or low-risk group. GSEA results showed a series of signaling pathway changes in the low-risk group including NF-κB pathway, regulation of autophagy, apoptosis, and JAK/STAT signaling pathways.

The role of *WARS* and *MGST1* in melanoma has not been reported. The *WARS* gene encodes tryptophanyl-tRNA synthetase, an aminoacyl-tRNA synthetase involved in protein synthesis and the regulation of RNA transcription and translation (26). *WARS* has been reported to be an IFN-γ-inducible enzyme, which protects indoleamine-2,3-dioxygenase expressing cells from tryptophan catabolism and mediates high-affinity tryptophan uptake into human cells (26, 27). Considering that tryptophan represents a powerful immunosuppressive mechanism hijacked by tumors for protection against immune destruction, *WARS* mediated tryptophan metabolism plays an essential role in immuno-oncology (28). *WARS* is dysregulated in different cancers with paradoxical roles on tumor invasiveness (29–34). In colorectal cancer, *WARS* was negatively correlated with lymph node metastasis and tumor stage, which could be explained by its antiangiogenic properties (31). Moreover, down-regulation of *WARS* by hypoxia could be a factor responsible for pancreatic cancer with high metastatic ability (35). However, in oral cancer, *WARS* is overexpressed and positively correlates with cancer invasiveness (32). Through bioinformatics analysis, we identified that *WARS* was a protective gene in melanoma. *WARS* prevents tumor cell progression, probably by inhibiting the neoangiogenic potential of the tumor (36). Further mechanism studies are needed to elucidate the paradoxical roles of *WARS* in tumors.

The *MGST1* gene encodes Microsomal Glutathione Transferase 1, a member of the *MAPEG* family (membrane associated proteins in eicosanoid and glutathione metabolism), which plays a well-established role in the conjugation of electrophiles and oxidative stress protection (37). The enzyme exhibits glutathione transferase and peroxidase activity, and shows activity against a variety of active substrates, from lipid peroxidation to cytostatic drugs (38). *MGST1* is overexpressed in various cancers (38, 39) and associated with drug resistance (37). Linnerth et al. suggested that overexpression of *MGST1* has been identified as an early marker in lung cancer (40). Further, Zeng and his colleagues demonstrated *MGST1* knockdown could inhibit lung adenocarcinoma cell proliferation by inactivating the AKT/GSK-3β pathway signaling and promote cell apoptosis by regulating the mitochondrial apoptosis pathway related proteins (39). Moreover, *MGST1* overexpression was correlated to higher metastatic potential in human prostate cancer (41). Surprisingly *MGST1* mRNA or protein cannot be detected in neuroblastoma cells or tissues (42). Here we reported that *MGST1* is a risk factor of melanoma and the detailed mechanism deserved further investigations. Our study provided not only a clinical tool for prognosis predictions but also the theoretical basis for future research studies.

After identifying the two metabolic prognostic genes, an MRGs signature was developed to predict the prognosis of melanoma patients. The MRGs signature was able to stratify OS in both training and validation cohorts and was a risk factor independent of clinicopathologic factors. We next established a nomogram for predicting 3- and 5-years OS based on MRGs signature, age, and stage. The ROC analysis and calibration plots were then applied to verify the prognostic accuracy, showing a good predictive performance of our model. Finally, the decision curve analyses in both training and validation datasets indicated that our model provided more clinical net benefits. Nomograms have been widely used in cancer management and prediction (43, 44). Several nomograms have been established for melanoma in recent years. Clinical and pathological features were applied to construct a nomogram to predict sentinel lymph node metastases in melanoma (45, 46). Nomograms were also developed to identify the risk, recurrence, and mortality in patients with negative sentinel lymph nodes (47, 48). There are two studies establishing models based on long non-coding RNA signatures to predict prognosis in melanoma patients (15, 49). To our knowledge, we conducted the first study to develop a nomogram to predict melanoma prognosis based on MRGs signature and clinicopathologic factors, exhibiting higher prognostic accuracy compared with the tumor-node-metastasis staging system.

Despite the potential clinical benefits of our results, our study has some limitations. We mainly focused on the effect of MRGs on melanoma prognosis; other genes, such as autophagy-related genes and immune-related genes, also contribute to the development and progression of melanoma. Additionally, our study was based on the whole population of melanoma patients, and the application to sub-populations still need investigated. Lastly, multicenter, large-scale prospective clinical trials are needed for further external validation of our nomogram.

In conclusion, a prognostic nomogram incorporating both MRGs signature and clinicopathological features for individual survival prediction was developed and validated, which is superior to the tumor-node-metastasis staging system.

## Data Availability Statement

The raw data supporting the conclusions of this article will be made available by the authors, without undue reservation, to any qualified researcher.

## Author Contributions

GD, XC, and FZ conceived and designed the study. JS and CP contributed to the outline development. FZ and GD analyzed the data and wrote the manuscript. ML, SZ, and YG revised the manuscript. All authors read and approved the final manuscript.

## Conflict of Interest

The authors declare that the research was conducted in the absence of any commercial or financial relationships that could be construed as a potential conflict of interest.

## Supplementary Material

Figure S1 Kaplan-Meier curves for disease free survival (DFS) of *WARS* **(A)** and *MGST1* **(B)** in melanoma patients using GEPIA. *N* (high) = 229, *N* (low) = 229. T, tumor; N, normal skin.

Figure S2 TCGA melanoma patients between high-risk and low-risk group based on the MRGs signature. Kaplan-Meier curves for DFS **(A)**, and the distribution of risk score, survival status and expression heatmap **(B)**.

Figure S3 Gene set enrichment and pathway analysis (GSEA). GO terms **(A)**, KEGG pathways **(B)**, and oncogenic signatures **(C)**.
