# Supplementary material for: Prognostic Implications of Metabolism Related Gene Signature in Cutaneous Melanoma
Source: Front Oncol. 2020 Sep 9;10:1710. doi: 10.3389/fonc.2020.01710 (PMC7509113; doi:10.3389/fonc.2020.01710)
Supplement: Supplementary file 1 [file Table_1.DOCX]

**Supplementary table 1. Clinical characteristics.**

OS, overall survival; yr, years; m, month.

|  | TCGA cohort (n = 460) | GEO dataset (n = 79) |
| --- | --- | --- |
| Age (Mean±SD, yr) | 58.1 ± 15.7 | 56.2 ± 15.1 |
| Gender (Percentage)  Male  Female | 286 (62.3%)  174 (37.7%) | 50 (63.3%)  29 (36.7%) |
| Stage (Percentage) |  |  |
| 0-Ⅱ | 232 (50.4%) | 58 (73.4%) |
| Ⅲ-Ⅳ | 192 (41.8%) | 20 (25.3%) |
| Unknown  OS (Mean±SD, m) | 36 (7.8%)  61.4 ± 64.5 | 1 (1.3%)  97.9 ± 47.3 |
