# Supplementary figures and images for: Prognostic Implications of Metabolism Related Gene Signature in Cutaneous Melanoma
Source: Front Oncol. 2020 Sep 9;10:1710. doi: 10.3389/fonc.2020.01710 (PMC7509113; doi:10.3389/fonc.2020.01710)

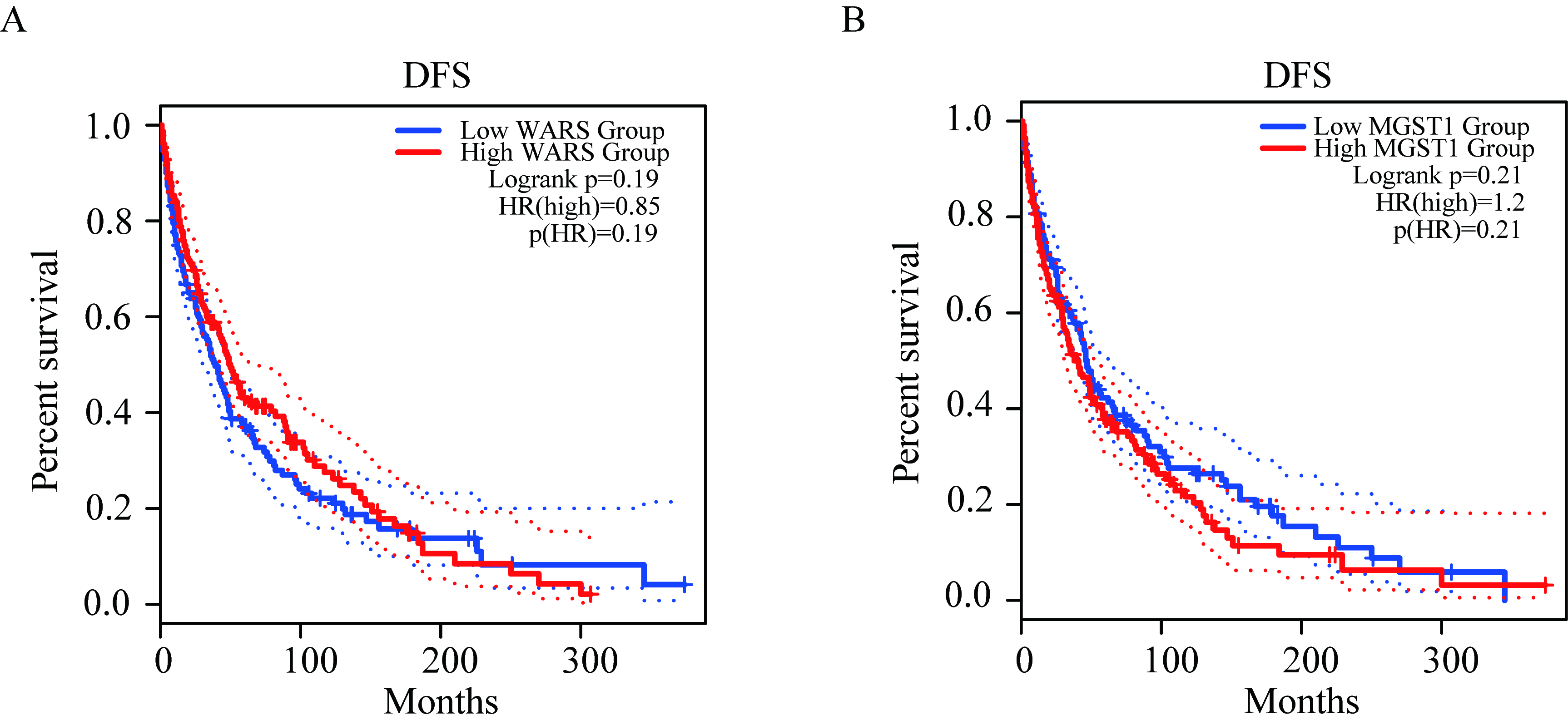

Supplement: Figure S1 — Kaplan-Meier curves for disease free survival (DFS) of WARS (A) and MGST1 (B) in melanoma patients using GEPIA. N (high) = 229, N (low) = 229. T, tumor; N, normal skin. [file Image_1.TIF]

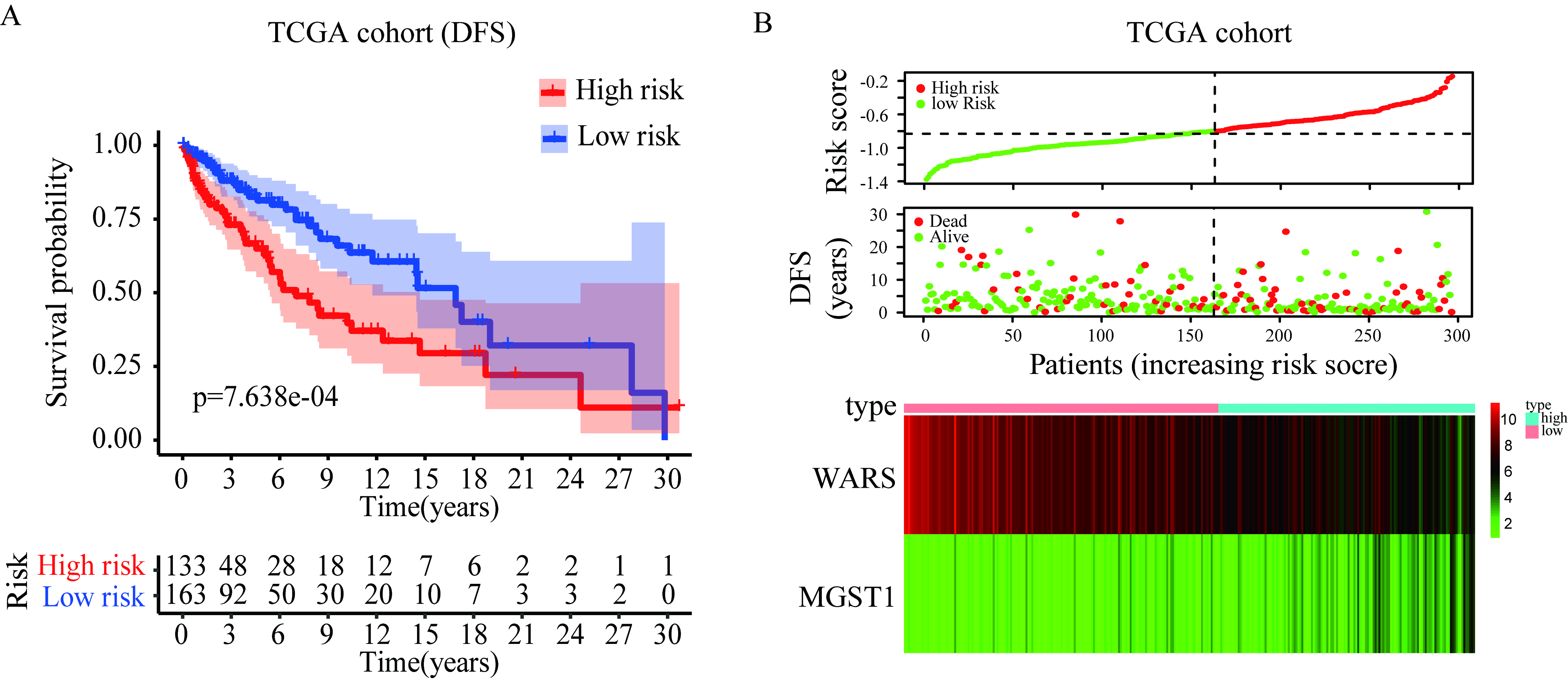

Supplement: Figure S2 — TCGA melanoma patients between high-risk and low-risk group based on the MRGs signature. Kaplan-Meier curves for DFS (A), and the distribution of risk score, survival status and expression heatmap (B). [file Image_2.TIF]

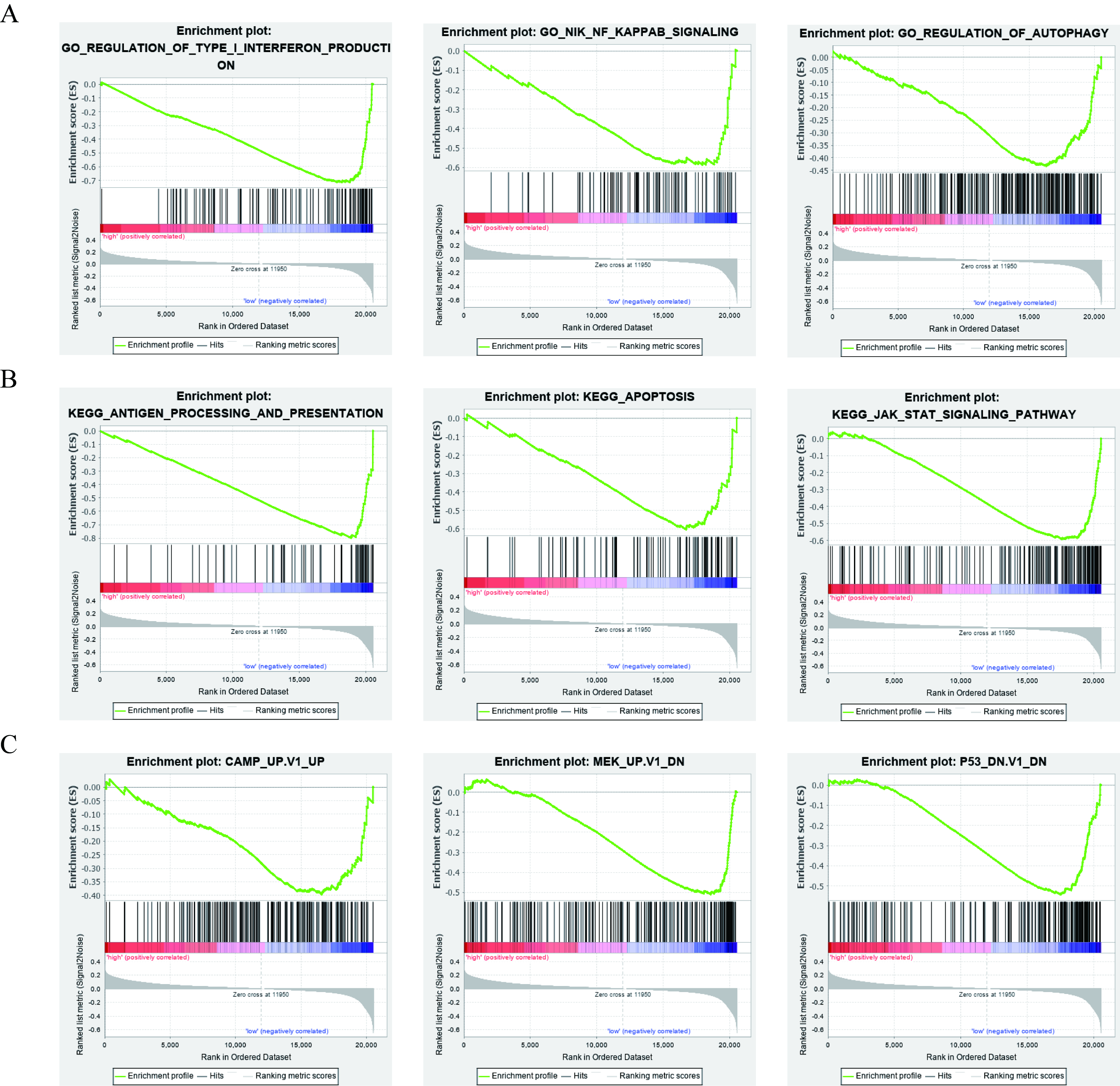

Supplement: Figure S3 — Gene set enrichment and pathway analysis (GSEA). GO terms (A), KEGG pathways (B), and oncogenic signatures (C). [file Image_3.TIF]
